# Novel small molecule modulators of quorum sensing in avian pathogenic *Escherichia coli* (APEC)

**DOI:** 10.1080/21505594.2018.1528844

**Published:** 2018-11-02

**Authors:** Yosra A. Helmy, Loic Deblais, Issmat I. Kassem, Dipak Kathayat, Gireesh Rajashekara

**Affiliations:** a Food Animal Health Research Program, Department of Veterinary Preventive Medicine, Ohio Agricultural Research and Development Center, The Ohio State University, Wooster, OH, USA; b Department of Animal Hygiene, Zoonoses and Animal Ethology, Faculty of Veterinary Medicine, Suez Canal University, Ismailia, Egypt; c Department of Nutrition and Food Sciences, Faculty of Agricultural and Food Sciences, American University of Beirut, Beirut, Lebanon

**Keywords:** APEC, autoinducer-2, quorum sensing inhibitors, virulence, biofilm, motility, infection, chicken, macrophages, wax moth model, gene expression

## Abstract

Colibacillosis caused by avian pathogenic *E. coli* (APEC), is an economically important bacterial disease of poultry. APEC are a subgroup of extra intestinal pathogenic *E. coli* (ExPEC) and poultry are considered potential sources of foodborne ExPEC to humans. Currently, APEC infections in poultry are controlled by antibiotics and/or vaccination; however, their effect is limited due to emergence of antibiotic resistant strains and infections with heterologous serotypes. Therefore, novel approaches are needed. Here, using the bioluminescent quorum sensing (QS) autoinducer 2 (AI-2) indicator *Vibrio harveyi* BB170, we screened the cell free culture supernatant of APEC O78 prepared from cultures grown in the presence of 4,182 small molecules (SMs; 100 μM). A total of 69 SMs inhibited > 75% of APEC O78 AI-2 activity in the indicator bacteria. Ten SMs that showed highest AI-2 inhibition were selected for further studies. Most of these SMs inhibited the AI-2 activity of other APEC serotypes and significantly reduced APEC O78 biofilm formation and motility. Most compounds showed minimal toxicity on human intestinal cells (Caco-2), chicken macrophage (HD-11), and chicken and sheep red blood cells, and reduced APEC survival in HD-11 and THP-1 macrophages. The SMs induced no or minimal toxicity and conferred protection against APEC in wax moth larval model. SMs affected the expression of APEC O78 QS, virulence, biofilm and motility associated genes providing insight on their potential mode(s) of action. Further testing in chickens will facilitate development of these SMs as novel therapeutics to control APEC in poultry and thereby also reduce zoonotic transmission.

## Introduction

Colibacillosis, caused by avian pathogenic *E. coli* (APEC), is a significant bacterial disease of poultry worldwide [1,2]. APEC belongs to a subgroup of extra-intestinal pathogenic *Escherichia coli* (ExPEC). APEC- like bacteria can be transmitted to humans through consumption of contaminated poultry and fresh produce contaminated with poultry litter [3]. Additionally, poultry ExPEC share many important traits with human ExPEC including antimicrobial resistance patterns, resistance genes, and virulence factors; thus, APEC pose a potential zoonotic risk for humans [4]. Even though there are several APEC serotypes implicated in avian colibacillosis, the most predominant serotypes associated with avian colibacillosis are O1: K1, O2: K1, and O78: K80 [5,6].

Avian colibacillosis is characterized by yolk sac infection, swollen-head syndrome, septicemia, and inflammation of different organs such as pericarditis, perihepatitis, airsacculitis, salpingitis, arthritis, and peritonitis [6,7]. APEC infects all ages of commercial poultry and can also negatively affect weight gain and feed conversion. Additionally, APEC infection is associated with high morbidity and mortality and carcass condemnation, leading to significant economic losses to the poultry industry [6].

Currently, APEC infections in poultry are controlled by a commercially available modified-live vaccine (Poulvac *E. coli*) [8]. However, the vaccination does not provide complete protection against all APEC serotypes, and high mortality in vaccinated broilers due to virulent APEC infections has been reported [8]. In addition, antimicrobials such as cephalosporins, tetracyclines, and quinolones that are used for treating APEC infections have limited effects due to the emergence of antimicrobial resistant strains [7]. Thus, there is a need for identifying novel approaches to enhance the control of APEC infections in poultry.

APEC possesses several virulence factors that have been determined to be involved in different stages of pathogenesis, including P fimbriae, type IV pili, curli, invasins, iron acquisition systems, serum complement resistance factors, anti-phagocytic factors, hemolysin E, outer membrane proteins A, and vacuolating autotransporter toxin [2,9,10]. Pathogenicity of APEC is also regulated by quorum sensing (QS) systems [2,11], which are mechanisms of bacterial cell-to-cell communication that involve density-dependent production, release and detection of extracellular signaling molecules called autoinducers (AIs) [12,13]. The QS autoinducer-2 signal molecule (AI-2) allows interspecies communication [14] and regulates expression of genes involved in various processes, including secretion of virulence factors, biofilm formation, motility, genetic competence, sporulation, and antibiotic production [12,15,16]. Furthermore, *luxS* which mediates the synthesis of AI-2 has also been shown to regulate motility, biofilm formation, virulence and pathogenesis of many bacterial pathogens, including APEC [2]. Therefore, inhibition of *luxS* and/or AI-2 activity using novel intervention such as QS small molecule inhibitors (QSI) can be a potential strategy for novel antibacterial development. As the QSI do not interfere with the metabolic processes of a bacterial cell such as protein synthesis, DNA metabolism, cell wall formation which are the targets for the development of drug resistance [17], they do not exert selection pressure on the bacteria during treatment, thus bacteria are less likely to develop resistance [17].

*Vibrio harveyi*, a marine Gram-negative bioluminescent bacterium, regulates its luminescence through QS [18]. *V. harveyi* regulates bioluminescence via two-component signaling systems, AI-1 and AI-2, each is composed of a sensor–autoinducer pair [18,19]. *V. harveyi* AI-2 indicator is capable of detecting the AI-2 of many bacteria that produce similar AI-2 molecules and stimulate light production following the addition of cell-free culture supernatants from these bacteria [20]. For example, AI-2 producing bacteria such as *E. coli*, *Salmonella typhimurium*, *Pseudomonas aeruginosa*, and *Vibrio cholerae* have been reported to produce signaling molecules that stimulate bioluminescence production in *V. harveyi* [19,20].

Here, we used *V. harveyi* BB170 AI-2 indicator bacteria (AI-1^+^ and AI-2^-^) [18] to identify small molecule AI-2 inhibitors of APEC. The selected AI-2 inhibitors were tested *in vitro* to evaluate their toxicity in human intestinal cells, chicken macrophages, chicken and sheep red blood cells (RBCs) and determine their efficacy against APEC infection in chicken and human macrophages and in wax moth (*Galleria mellonella*) larva model. Further, the expression of several QS, virulence, biofilm and motility associated genes were assessed to provide insight on their potential mode(s) of action on APEC O78. We identified 10 small molecules (SMs) that modulated APEC infection *in vitro* and *in vivo*. These SMs can serve as leads for future application in APEC control in poultry and thereby also reduce potential zoonotic transmission of APEC to humans.

## Results

### Primary screening identified 69 compounds that significantly inhibited AI-2 activity of APEC

**Figure 1.**
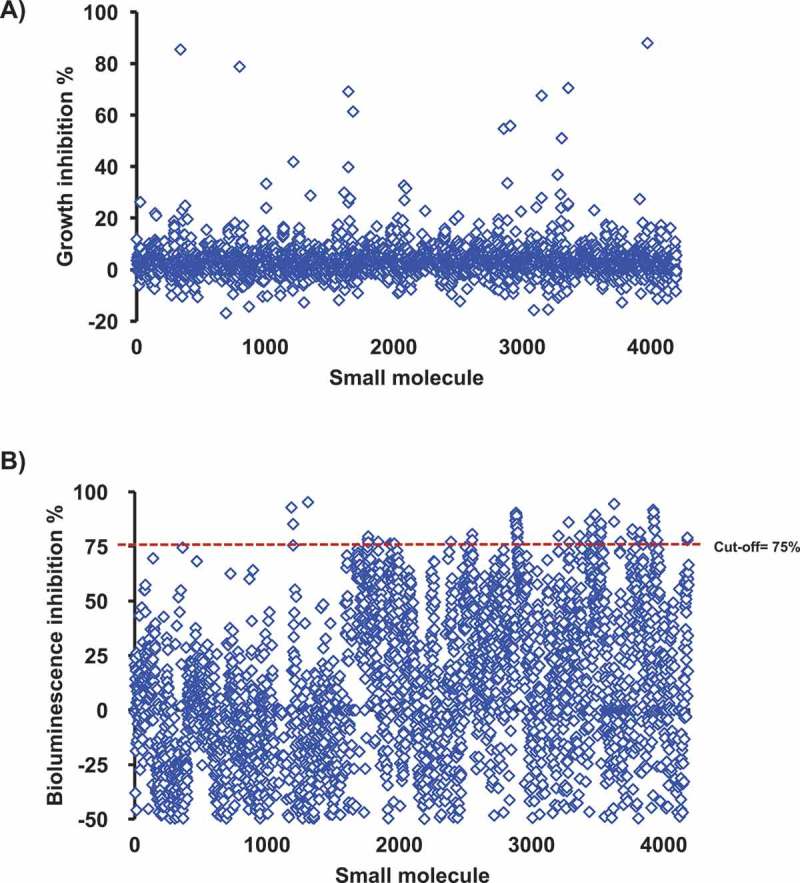
A) High-throughput screening for growth inhibition against APEC O78. 4,122 compounds did not impact the growth of APEC O78 (no elevated OD). B) Screening of the SMs for their effect on the AI-2 activity of APEC O78. The *Vibrio harveyi* BB170 AI-2 bioluminescent indicator bacteria was used to screen the cell-free culture supernatant of APEC O78. APEC cell-free culture supernatant was prepared from cultures grown in the presence of 100 μM of small molecules. Sixty nine compounds inhibited ≥ 75% of the AI-2 activity of APEC O78. AI-2 bioluminescence indicator assay was repeated four times for these 69 compounds and 10 compounds that showed highest AI-2 inhibition were selected for further studies.

A library of 4,182 small molecules (SMs) was screened for APEC O78 growth inhibition. The results showed that 4,122 molecules did not impact the growth of APEC O78 (no elevated OD) (Figure 1A). Supernatants from cultures grown in the presence of 4,122 compounds were screened for their ability to inhibit bioluminescence of the *V. harveyi* AI-2 indicator. Inhibition of bioluminescence by SM treated APEC O78 culture supernatants was compared to that of the DMSO treated control. A total of 69 compounds inhibited ≥ 75% of the bioluminescence activity. These 69 compounds were further tested for AI-2 mediated bioluminescence inhibition in four independent experiments and 10 compounds (referred hereafter as AI-2 inhibitors) that showed highest AI-2 inhibition (75–98%) were selected for further studies (Figure 1B). However, our analysis revealed that only 25% of AI-2- associated bioluminescence inhibition was achieved with some AI-2 inhibitors (C3- C5, C7, C9 and C10) at 50 µM and no inhibition was observed at lower concentrations (data not shown).

Further, the selected AI-2 inhibitors displayed differential effect on the inhibition of AI-2 activity of multiple APEC serotypes. Most compounds resulted in ≥ 75% inhibition of AI-2 activity of APEC O2 with the exception of C3 and C9, while only C1 and C6 resulted in ≥ 75% inhibition of AI-2 activity of APEC O1. Only C6 exhibited ≥ 75% inhibition of AI-2 activity of O8, O15, O18, and O35. Whereas C8 exhibited ≥ 75% inhibition of AI-2 activity of O109, while C6 and C10 exhibited ≥ 75% inhibition of AI-2 activity of O115 (Table 1).

**Table 1.** Effect of the selected SMs on the AI-2 activity of different APEC serotypes.

	% Inhibtion										
Serotypes	C1	C2	C3	C4	C5	C6	C7	C8	C9	C10	Sources
O78	84	92	78	75	83	92	94	98	79	84	[62]
O2	94	96	54	76	89	93	83	94	51	89	[63]
O1	76	64	65	63	46	93	47	41	19	64	[4]
O8	20	20	39	0	18	79	2	18	20	0	[63]
O15	13	27	0	46	45	97	36	25	0	22	[63]
O18	37	43	8	54	61	95	48	54	14	35	[63]
O35	48	55	37	60	68	96	62	57	36	47	[63]
O109	65	57	0	67	69	60	67	75	66	57	[63]
O115	64	52	35	58	63	99	45	44	44	78	[63]

Inhibition of AI-2 activity was calculated by comparing bioluminescence inhibition from SM treated APEC O78 culture with that of the DMSO treated control

### The selected AI-2 inhibitors affected biofilm formation and motility of APEC O78

**Figure 2.**
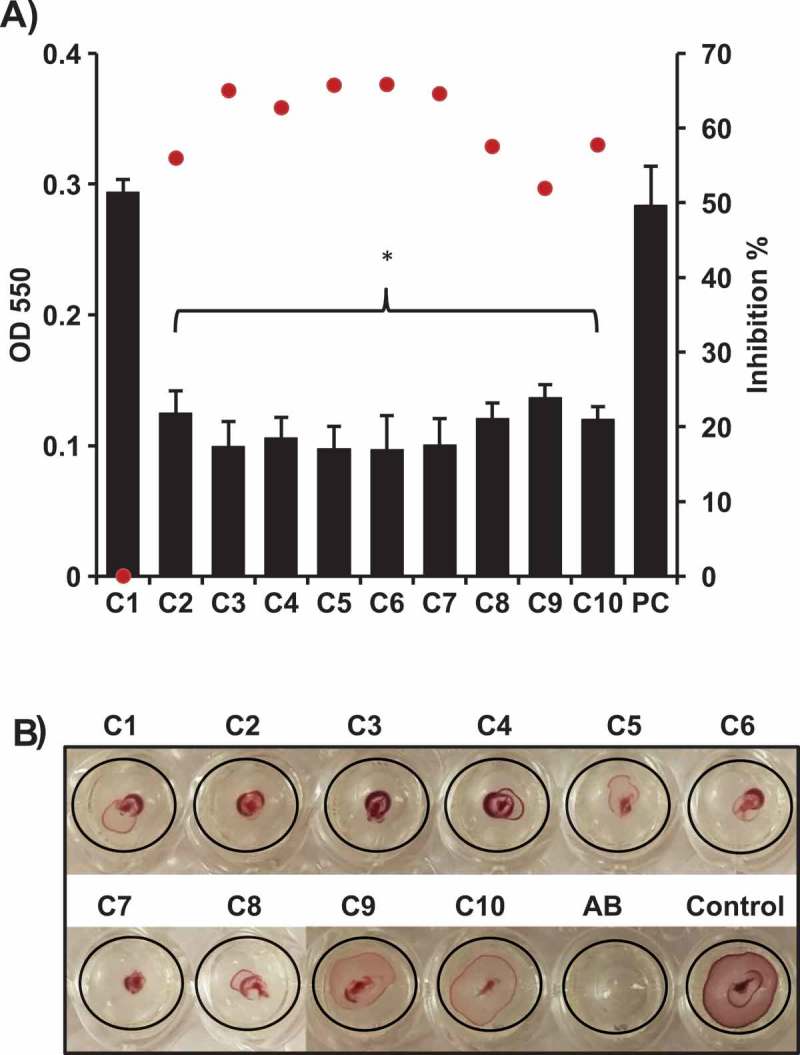
Effect of the selected AI-2 inhibitors on; A) biofilm formation of APEC O78. Biofilm formation was assessed using crystal violet assay by measuring the OD_550_. All of the compounds significantly inhibited the biofilm formation except C1 in comparison to the DMSO treated control (PC). Two independent experiments were conducted with triplicate wells in each experiment and the average OD_550_ (black bars) and inhibition % (red dots) are shown. B) motility of APEC O78. Motility was assessed by measuring the halo on a semisolid agar. Except C9 and C10, all compounds resulted in complete inhibition (did not form detectable motility halos) in comparison to the DMSO treated control. *Significant difference between AI-2 inhibitors treated compared to DMSO treated control (P < 0.05).

Biofilms play a crucial role in APEC virulence and enhance the bacterial resistance to antimicrobials and immune clearance, leading to failure of antimicrobial therapy [21,22]. Therefore, quorum sensing inhibitors have been proposed as promising anti-biofilm agents [21,22]. The effect of the AI-2 inhibitors on APEC O78 biofilm formation was determined using the crystal violet staining (CV) assay. Biofilm formation was assessed after 48 h of incubation in the presence of 100 µM of each compound. We noted that C3 – C7 resulted in 63–66% reduction of APEC O78 biofilm formation, while C2, and C8 – C10 resulted in 52–58% reduction of APEC O78 biofilm formation (P < 0.05) (Figure 2A).

Motility and chemotaxis allow bacteria to migrate towards or away from favorable environments in response to stimulus and thus contribute to bacterial fitness and virulence [23]. The effect of the AI-2- inhibitors on motility of APEC O78 was determined using 100 µM of each compound. With the exception of C9 and C10, all compounds resulted in inhibition (did not form detectable zones of motility) of APEC O78 motility after 6 h of incubation in comparison to the DMSO treated control (Figure 2B). Notably, the diameter of the zone of motility was reduced up to 75% in comparison to DMSO treated control (from 8 mm in DMSO treated control to 2 mm on an average in AI-2 inhibitors treated wells).

### The AI-2 inhibitors showed low toxicity on human colonic adenocarcinoma epithelial cells (Caco-2) and chicken macrophage cells (HD-11) and no hemolytic activity on sheep and chickens red blood cells (RBCs)

**Figure 3.**
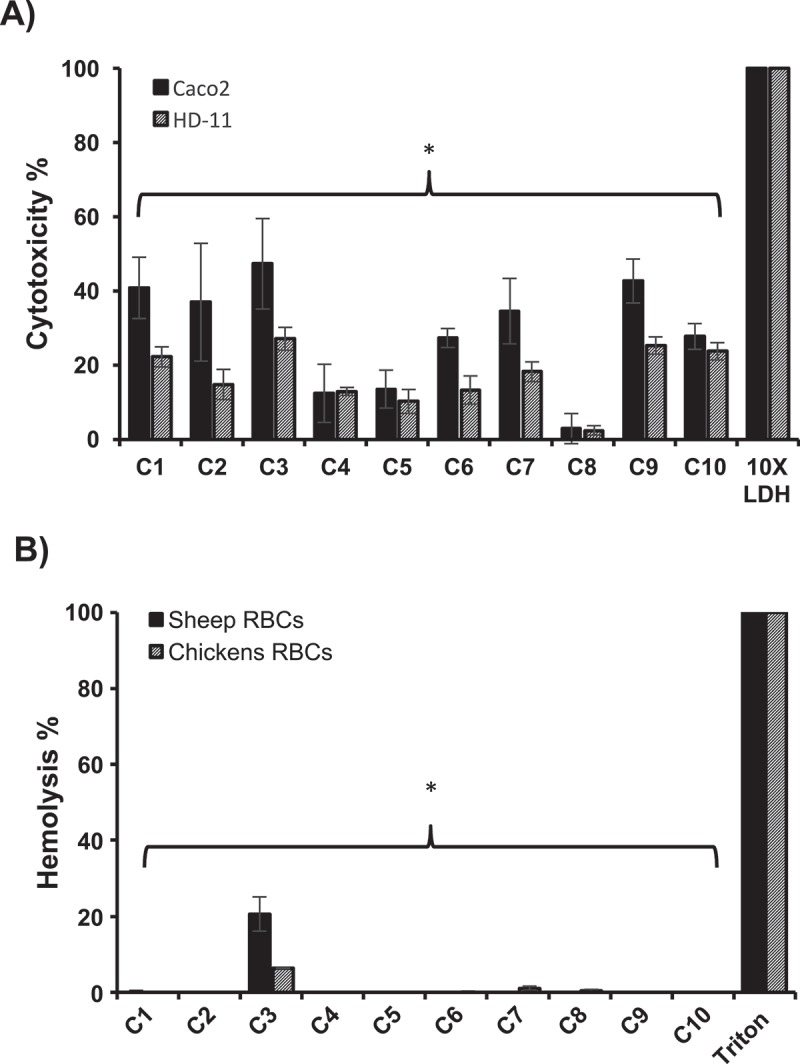
Cytotoxicity (A) and hemolytic activity (B) of the selected AI-2 inhibitors. Cytotoxicity was assessed using Caco-2 and HD-11 cells and hemolytic activity was determined using sheep and chicken RBCs. 100 μM of each compound was used in both assays. Most of the compounds showed significantly less cytotoxicity and hemolytic activity (P ≤ 0.05) compared to DMSO treated control. Two independent experiments were conducted with triplicate wells in each experiment and the average is shown. *Significant difference between AI-2 inhibitors treated wells compared to 10X LDH (cytotoxicity assay control) or Triton X-100 (hemolytic activity assay control).

The toxicity of the selected AI-2 inhibitors was assessed on Caco-2 and HD-11 cells using lactate dehydrogenase (LDH) assay to quantitatively measure the LDH released into the media from damaged cells after exposure to AI-2 inhibitors as an indicator for cellular cytotoxicity and cytolysis. Our results showed that, when treated with 100 µM of SMs, C8 and C4 exhibited 2% and 12% cytotoxicity, respectively; while C6 and C10 exhibited 27% cytotoxicity and C1-C3, C7, and C9 displayed 36% to 42% cytotoxicity on Caco-2 cells. Further, in HD-11 cells, C5 and C8 exhibited less than 10% cytotoxicity, while C2, C4, C6, and C7 exhibited cytotoxicity between 12% and 18%, and C1, C3, C9 and C10 exhibited toxicity between 22% and 27% (Figure 3A).

Additionally, toxicity of the selected AI-2 inhibitors to sheep and chicken RBCs was also tested using 100 µM of each compound. Notably, all compounds exhibited no hemolytic activity against sheep or chicken RBCs with the exception of C3, which exhibited 20.6 % and 6.4% hemolytic activity in sheep and chicken RBCs, respectively (Figure 3B).

### The selected AI-2 inhibitors reduced the survival of APEC O78, O2, and O1 in chicken and human macrophage cells

**Figure 4.**
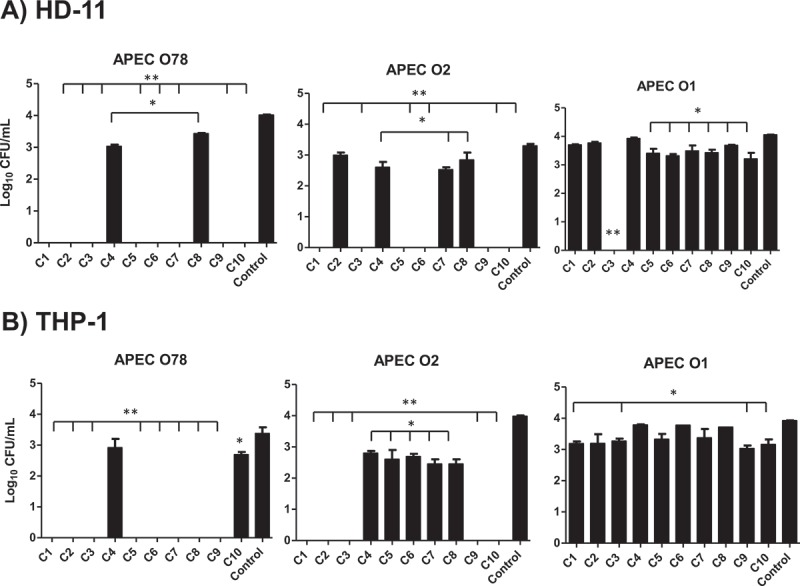
Effect of the AI-2 inhibitors on the intracellular survival of different APEC O78, O2, and O1 in HD-11 (A) and THP-1 (B) cells. Cells were infected with APEC strains at MOI = 100, treated for 6 h with 1 µL (100 µM) of each compound and the internalized bacteria were determined. Two independent experiments were conducted with triplicate wells in each experiment and the average is shown. *Significant difference between AI-2 inhibitors treated cells (P < 0.05) compared to DMSO treated control. **Significant difference between AI-2 inhibitors treated cells (P < 0.001) compared to DMSO treated control.

The invasion of APEC into phagocytic cells facilitates its intracellular survival and systemic spread to different organs which is critical for APEC pathogenesis [24]. Therefore, the impact of the AI-2 inhibitors on APEC O78, O1 and O2 (the most predominant serotypes associated with colibacillosis) survival in HD-11 and acute human leukemia macrophage (THP-1 cells) was assessed using 100 µM of each compound. In HD-11 cells, treatment with C1-C3, C5-7, C9 and C10 resulted in 100% clearance of APEC O78 (~ 4 logs reduction; P < 0.001), while C4 and C8 significantly reduced its intracellular survival (up to 1 log; P < 0.05) when compared to DMSO treated control. Additionally, C1-C3, C5, C6, C9 and C10 resulted in 100% clearance of APEC O2 (~ 3.3 logs reduction; P < 0.001), and C4, C7 and C8 significantly reduced its intracellular survival (up to 0.8 log; P < 0.05). Only C3 exhibited 100% clearance of APEC O1 (~ 4 logs reduction; P < 0.001), while C5- C8 and C10 significantly reduced its intracellular survival (up to 0.9 log; P < 0.05) (Figure 4A).

Similarly, in THP-1 cells, C1- C3, and C5- C9 exhibited 100% clearance of APEC O78 (~ 3.4 logs reduction; P < 0.001), while C10 significantly reduced its intracellular survival (up to 0.7 log; P < 0.05). C1- C3, C9 and C10 exhibited 100% clearance of APEC O2 (4 logs reduction; P < 0.001), and C4- C8 significantly reduced its intracellular survival (up to 1.5 logs; P < 0.05). Similarly, C1, C3, C9 and C10 significantly reduced the intracellular survival of APEC O1 (up to 0.9 log; P < 0.05) (Figure 4B). Interestingly, we observed varying effects of the AI-2 inhibitors on different internalized APEC serotypes in HD-11 and THP-1 cells. Specifically, some AI-2 inhibitors were less effective against O1 and O2. This variation might be due to differences in their capsule composition. Both the O1 and O2 possess K1 (sialic acid) containing capsule, which confers protection against host immunity and increase resistant to the bactericidal effect of serum as compared to O78 which expresses different capsular antigens [25,26]. In addition, O1 also contains pathogenicity island (PAI I_APEC-O1_) that carries *pap* operon (putative virulence genes of APEC), invasion determinant protein encoding gene *tia* and iron-regulated outer membrane protein encoding gene *ireA* which are likely to contribute to increased virulence of APEC O1; thus, the lower efficacy of SMs [27].

### The AI-2 inhibitors increased the survival of APEC infected wax moth (Galleria mellonella) larvae

**Figure 5.**
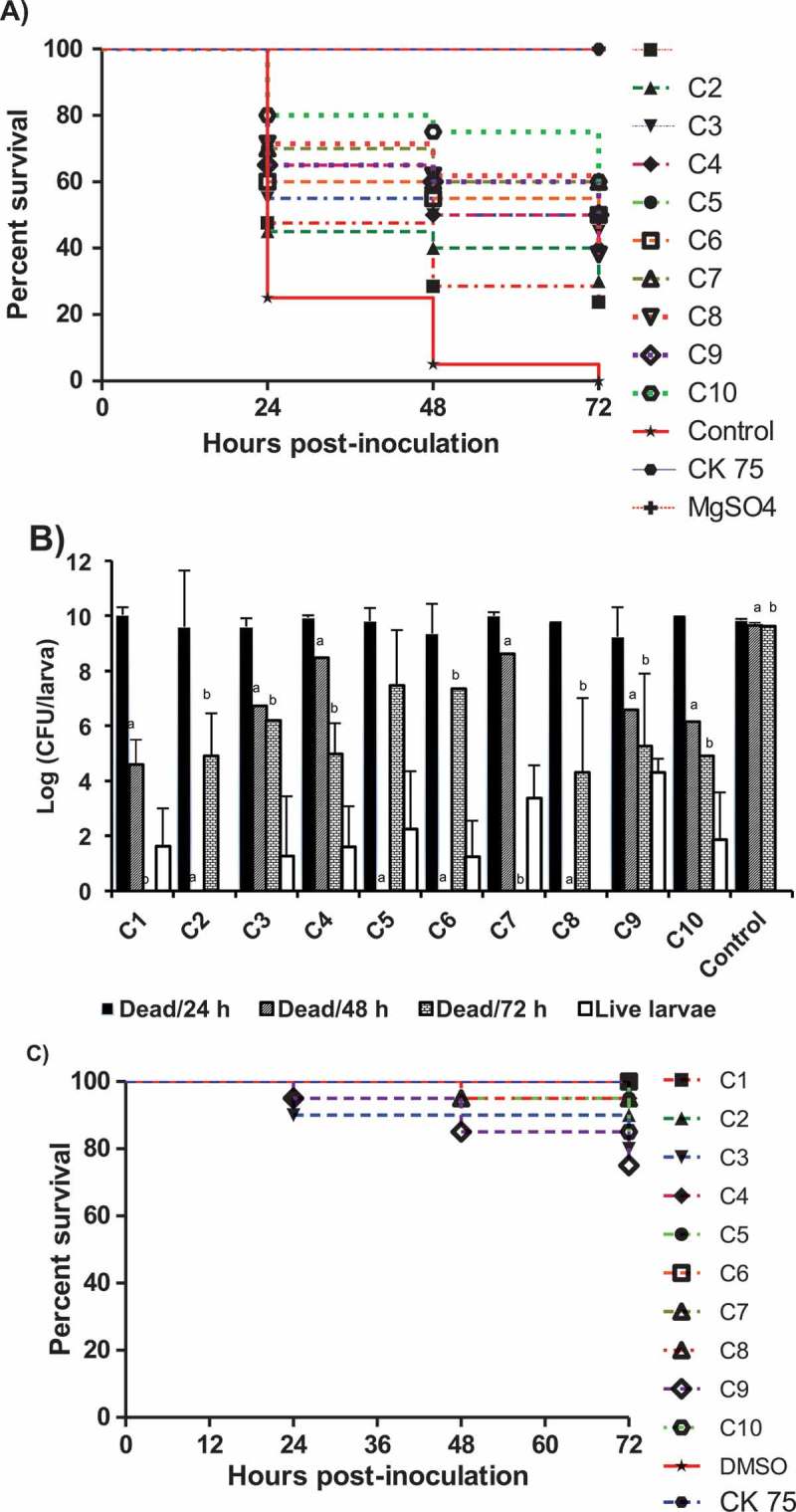
Effect of the AI-2 inhibitors on *G. mellonella* larvae infected with APEC O78; A) survival rate of the treated larvae, B) bacterial load in dead larvae at different time points (24, 48, 72 h) and in live larvae at 72 h, and C) toxic effect of each compound on *G. mellonella* larvae. The larvae were treated with the AI-2 inhibitors (12.5 µg/larva) and inoculated with APEC O78 (8.5 µL ~ 4.25 × 10^4^ CFU), survival monitored for 72 h. The results of two independent experiments were averaged. Significant difference between AI-2 inhibitors treated compared to DMSO treated control at 48 h^a^ and 72 h^b^ (P < 0.05).

The *in vivo* efficacy of the AI-2 inhibitors was evaluated using the wax moth larva model, which has been previously used to evaluate drug efficacy and bacterial pathogenesis [28]. Treatment of the infected larvae with C1 and C2 resulted in 23% and 30% increase in larval survival, respectively; while compounds C3-10 resulted in 40–60% increase in larval survival in comparison to DMSO treated controls (Figure 5A).

Treatment of the wax moth larvae with the AI-2 inhibitors also reduced APEC O78 colony forming units (CFU) in both dead and live larvae in comparison to DMSO control. At 72 h post treatment, the bacterial load in dead larvae in all treated groups, except those treated with C1 and C7 that showed no larval death, were significantly reduced (~ 4.3–7.5 logs; P < 0.05) in comparison to DMSO treated control. However, in surviving larvae, C2 and C8 resulted in 100% clearance of APEC (up to 5 logs; P < 0.001), while C4-C6 and C10 significantly reduced the bacterial load (up to 3.5–6 logs; P < 0.05) in comparison to dead larvae. Further, at 48 h post treatment, C2, C5, C6 and C8 treated groups showed no larval death while the rest of the compounds resulted in significant reduction of the APEC O78 load (1.2–3.5 logs; P < 0.05) in comparison to DMSO treated controls (Figure 5B). However, the larvae died at 24 h post treatment possessed APEC load similar to DMSO treated controls. These results indicated that the bacterial load in all AI-2 inhibitors treated groups, except C9, significantly decreased over time (r ≤ −0.87; p ≤ 0.001) in comparison to DMSO treated control.

Notably, all AI-2 inhibitors showed no or low toxicity in larvae. The survival of larvae treated with C1 and C6 was 100%, whereas the survival of larvae was more than 75% for the rest of the AI-2 inhibitors (Figure 5C). These results suggest that the larval death is not due to the toxic effect of AI-2 inhibitors but rather due to APEC O78 infection. Thus, the wax moth model may serve as rapid screening tool to assess the efficacy of potential antimicrobials against APEC and for selection in *in vivo* studies in the natural host.

### The QS AI-2 inhibitors affected the expression of virulence, biofilm formation and motility- associated genes of APEC

Quorum sensing inhibitors have been proposed to affect the expression of QS regulated and virulence- associated genes [17]. Therefore, to elucidate how the AI-2 inhibitors might attenuate APEC pathogenicity [2,13], we assessed the expression of genes representing multiple physiological processes regulated by QS (Table 2) such as AI-2 synthesis (2 genes), small molecules metabolism (5 genes), virulence factors (6 genes), biofilm formation, cell motility and exopolysaccharide formation (11 genes), cell division, DNA processing, and morphology (5 genes).

**Table 2.** Real Time PCR Primers used in this study.

Function	Genes	Gene product	Oligonucleotide sequence (5’ to 3’)	product size (bp)
AI-2 synthesis, biofilm formation, cell motility and exopolysaccharide formation	*luxS*	S-ribosylhomocysteinase	F: ACGAGTGCATCTGGTAAGTG R: CAATGGAAGACGTGCTGAAAG	87
	*pfs*	5’-methylthioadenosine/S-adenosylhomocysteine nucleosidase	F: CGGCAGAACCGGTGTTAATAAT R: TGAAATCGGGCATCGGTAAAG	96
	*hha*	Hemolysin expression-modulating protein	F: GTGATCTGCGGCTGAGTAAA R: ACGTCGTTGCCAGACAAT	103
	*wzb*	Protein-tyrosine-phosphatase	F: TCGTTATCCCAGTGACCAAAC R: GTGTCGCAACTATGACCTGAT	114
	*ompG*	Outer membrane protein G precursor	F: CGGTTGGCTGTCGATGTATAA R: AGGTATATTGCAGACCCGTTTC	95
	*rcsB*	Transcriptional regulator RcsB	F: CAAGTACATCAAGCGCCATTTC R: CCCTTCGATATCCAGATCCAATAC	103
	*motB*	Flagellar motor protein B	F: AGGCTAATACGGTTGGGAATAC R: AGAATCGCCCGATGTTTAGAA	109
	*flgN*	Flagella synthesis chaperone protein FlgN	F: CCAGTAACCAGCCGTTATGTT R: TACGCAGGAAAGAACCCAATAC	117
	*fliP*	Flagellar biosynthetic protein FliP	F: AGCCATTCAGCGAAGAGAAA R: GTCTGGCAAACAACCCTAAATC	117
	*cheW*	Purine-binding chemotaxis protein CheW	F: CATCCACCTGGCTGAACTTA R: GTAACACGGATTGCGAACAC	106
	*nlpC*	Probable lipoprotein NlpC precursor	F: GCATCGTCACAACCACAAATC R: GGTTTGAACGACCAGCTACA	103
Virulence- associated genes	*ompA*	Membrane protein	F: TGACCGAAACGGTAGGAAAC R: GGAATACCAGTGGACCAACAA	99
	*fimC*	Fimbrial chaperone protein	F: GCTGGCAGGTATCCTGATATTC R: TGCCCTGCCGGATAAATTAC	99
	*IucD*	IucD protein	F: GTCCGGAGAAGCCTGAAATA R: GAGAAGCGGCGGAAATAAAC	114
	*fyuA*	Ferric yersiniabactin uptake A	F: CTTCCCTTCCGGTTCGTTAATC R: GGTACAGCCCAAACACCATATC	119
	*iss*	Serum survival protein	F: CGCTCTGGCAATGCTTATTAC R: GAAATGATGGGTGATGGTTTCC	100
	*vat*	Vacuolating autotransporter toxin	F: CTGAACCGCGTCCAGATTAT R: ACTCCACGGCAGGAAATATG	104
Cell division, DNA processing, and morphology	*bolA*	BolA protein	F: CAGTACTTTAGCGGAGGAACTC R: CAACCCTTCCCACTCCTTAAT	82
	*mreD*	Rod shape-determining protein MreD	F: CCGGTCTGAAAGAGACGTTAAT R: TTCCGCAACCTCGCATTAT	115
	*csrA*	Carbon storage regulator A	F: GTAACTGGACTGCTGGGATTT R: CCAGGTACGTATTGGCGTAAA	100
	*murD*	UDP-N-acetylmuramoylalanine- D-glutamate ligase	F: GCGTGGTTAATGCTGATGATG R: CCTGCTGATGATTCAGGTGATA	108
	*lpp*	Major outer membrane lipoprotein precursor	F: CGGTAATCCTGGGTTCTACTCT R: TGCTCAGCTGGTCAACTTTAG	108
Small molecules metabolism	*frwC*	Fructose-like-2 IIC component	F: AGCAGGGCAGCATTGTTAT R: GCAAATCAGCATGAAGGCATAG	104
	*Fpr*	Ferredoxin-NADP reductase	F: GTTCCTGCATCAGAGGTAGATAG R: TACAGCGATTGGCCCTTATT	128
	*mtlR*	Mannitol operon repressor	F: CGTCATTAACCGCCAGGAATA R: GTTCACCAAAGGGTCCAAGTA	122
	*ugp*C	SN-glycerol-3-phosphate transport ATP-binding protein UgpC	F: CTGATCGTGGGTAACGTAGAG R: GCAGTGTTCCTGTTTGATGAG	126
	*thiH*	Thiazole biosynthesis protein ThiH	F: GCATAAGTCGCCTCGTGATATG R: GACGGAATACGCCGAGTTAAAG	81
Housekeeping gene	*GAPDH*	Glyceraldehyde-3-phosphate dehydrogenase	F: CGGTACCGTTGAAGTGAAAGA R: ACTTCGTCCCATTTCAGGTTAG	99

**Figure 6.**
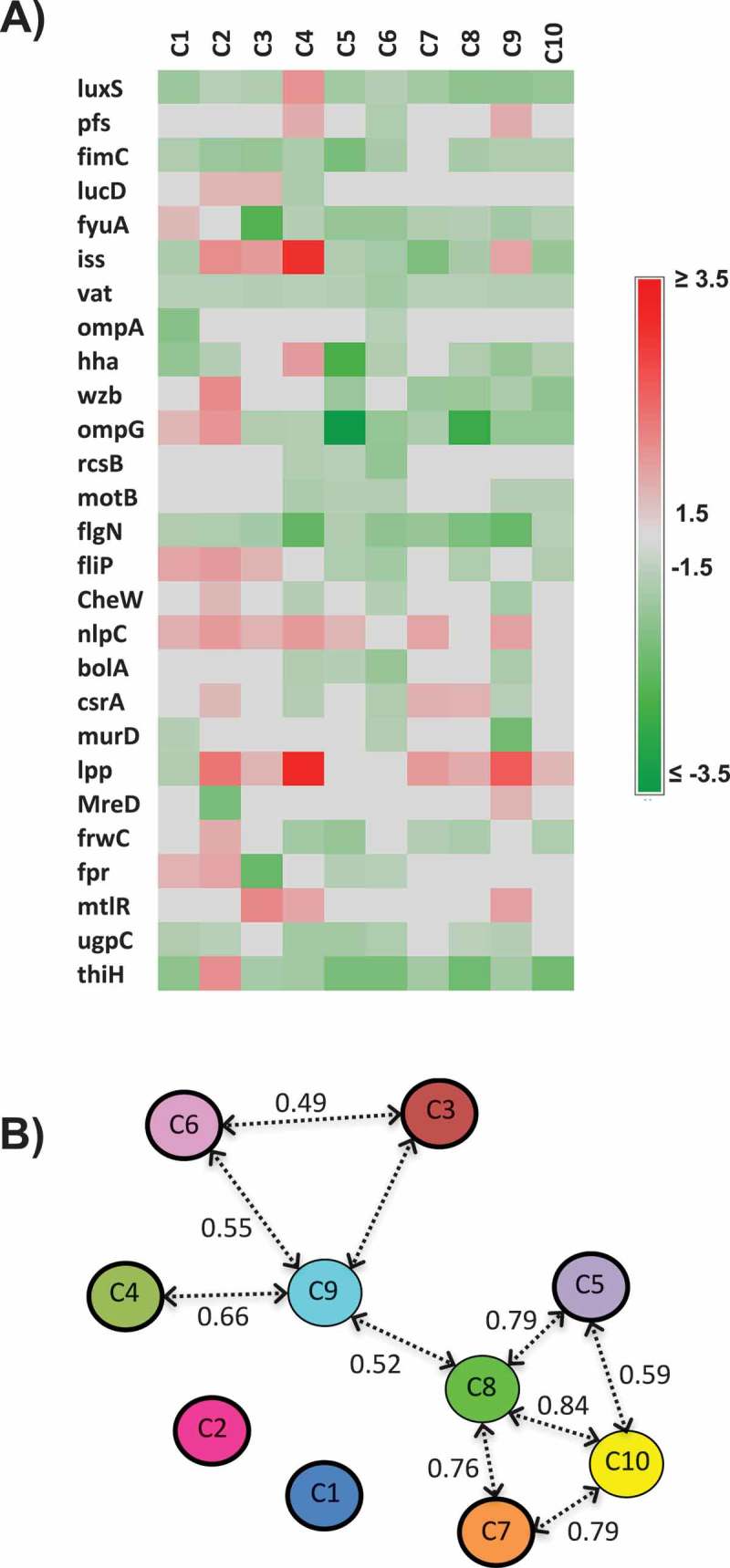
Effect of the AI-2 inhibitors on expression of QS and virulence-associated genes; A) Heat map showing the impact of the selected AI-2 inhibitors on gene expression of QS and virulence associated genes. Effect on gene expression was assessed using 100 µM of AI-2 inhibitors. Three independent experiments were conducted and the average fold change was calculated using ∆∆ct. Fold changes ± 1.5 ≥ or ≤ 1.5 were considered differentially expressed. B) Principal component analysis (PCA) of the qRT-PCR data. Numbers above the arrows indicate the correlation (r) between the AI-2 inhibitors based on the gene expression data. The PCA analysis is based on the fold change of gene expression data.

Interestingly, all compounds, with the exception of C4, down-regulated the expression of cytosolic S-ribosylhomocysteine lyase gene (*luxS*), which mediates AI-2 synthesis. Notably, C4 up-regulated the expression of 5’-methylthioadenosine/S-adenosylhomocysteine nucleosidase gene (*pfs*), which also mediates AI-2 synthesis (Figure 6A). Further, AI-2 inhibitors affected group of genes that influence AI-2 production and degradation and small- molecules metabolism. These genes have not been directly implicated in cell- cell communication but provide a link between QS and central metabolism [13]. The AI-2 inhibitors down-regulated genes encoding inner membrane sugar transport fructose-like-2 IIC component (*frwC*; in response to C5- C8 and C10), carbohydrate transport sn-glycerol-3-phosphate import ATP-binding protein (*ugpC*; C1, C2, C4- C6, C8 and C9), cytoplasmic ferredoxin-NADP reductase (*fpr*; C3, C5 and C6), and thiazole biosynthesis protein (*thiH*; C1 and C3- C10) (Figure 6A).

Since the expression of virulence factors by APEC is among many traits controlled by QS [29], inhibition of these virulence factors by QSI might attenuate APEC virulence. Notably, AI-2 inhibitors down-regulated the expression of genes encoding periplasm fimbrial chaperone protein *fimC*; C1- C6, and C8- C10), iron uptake chelate protein D (*iucD*; by C4), ferric yersiniabactin uptake A (*fyuA*; by C3- C10), serum survival protein (*iss*; by C1, C5- C8, and C10), and vacuolating autotransporter toxin (*vat*; by C1-C10). However, C2- C4 and C9 up-regulated the expression of *iss* (Figure 6A).

AI-2 inhibitors also affected the expression of biofilm formation, motility and exopolysaccharide formation associated genes. The AI-2 inhibitors down-regulated the expression of genes encoding the hemolysin-expression modulating protein (*hha*; C1, C2, C5, C6 and C8- C10), transcriptional regulator protein (*rcsB*; C4- C6), outer membrane protein G precursor (*ompG*; by C2- C10), and tyrosine-phosphatase protein (*wzb*; by C5, and C7- C10). Both *hha* and *rcsB* are located in the cytosol and were shown to be involved in biofilm formation, while *ompG* and *wzb* contributes to exopolysacchride formation. Likewise, genes associated with chemotaxis and motility, such as cytoplasmic flagellar synthesis chaperone protein (*flgN*; C1-10), chemotaxis protein (*cheW*; C4, C6, and C9), flagellar motor protein B (*motB*; by C4- C6, and C9- C10) and flagellar biosynthesis protein (*fliP*; by C5, C6, C8, and C10) were also down-regulated by AI-2 inhibitors (Figure 6A). However, C1-C5, C7, and C9 up-regulated the expression of cell membrane probable endopeptidase (*nlpC*).

Quorum sensing has also been implicated in the regulation of multiple physiological processes such as DNA replication, cell division and cell morphology [30]. The AI-2 inhibitors down-regulated the expression of genes encoding transcriptional regulators such as cytosolic DNA-binding transcriptional regulator (*bolA*; C4- C6, and C9), and carbon storage regulator A (*csrA*; C4, C6, and C9), and UDP-N-acetylmuramoylalanine- D-glutamate ligase (*murD*; C1, C6, and C9). However, C2-C4, and C7- C10 up-regulated the expression of major outer membrane lipoprotein precursor gene (*lpp*) (Figure 6A).

Principal component and multivariate analysis were performed to determine the gene expression profiles of APEC treated with the AI-2 inhibitors. The expression profiling divided the AI-2 inhibitors into 2 groups (r > 0.49; P < 0.01). The first group composed of C5, C7, C8 and C10 (0.59 ≤ r ≤ 0.84), while the second group composed of C3, C4, C6 and C9 (0.49 ≤ r ≤ 0.66). The C9 and C8 showed high correlation with other AI-2 inhibitors (r = 0.52; P = 0.005) and they connected these two groups together (Figure 6B). Further, C5 to C10 displayed similarity in their expression profile (r > 0.4; P < 0.05), while C1 and C2 showed no significant expression profiles similarity with the other AI-2 inhibitors (r < 0.33) (Figure 6B). Despite the high similarity scores between certain AI-2 inhibitors, the gene expression profiles differed between AI-2 inhibitors, suggesting that each AI-2 inhibitors might interact with different target(s) that potentially share common biological pathways within APEC.

## Discussion

The increase in antibiotic resistant APEC [7], the limited effect of current vaccine [8], and the ability of APEC to establish persistent infections through the formation of biofilms [2] emphasize the need for alternate control strategies for APEC. A great effort has been made to develop anti-pathogenic drugs by reducing the bacterial virulence through QSIs, which proves to be an intriguing approach for antimicrobial chemotherapy [31]. In this study, we identified, using *V. harveyi* BB170 AI-2 indicator bacteria, 10 novel QS AI-2 inhibitors that did not inhibit APEC growth but interfered with QS- regulated processes including virulence factors, biofilm formation, motility, exopolysaccharide synthesis, stress survival, cell division and pathogenesis in APEC [13]. Use of QSI to attenuate APEC pathogenicity is attractive as this approach is less likely to result in the development of resistant APEC.

All 10 AI-2 inhibitors except C4, attenuated the AI-2–production and down-regulated the expression of *luxS*. Further, most of the SMs also down-regulated the expression of QS- regulated and virulence-associated genes such as *pfs, iucD, fyuA, vat, ompA, iss*, and *fimC* (Figure 6A). Similarly, APEC *luxS* deletion mutant has been reported to affect AI-2 activity and down-regulate virulence-associated genes [2,32]. In addition, deletion of *luxS* also resulted in reduced adherence and invasion abilities of APEC O78 and, consequently, its survival in cultured cells [2,5]. Adherence and invasion are important for APEC pathogenesis and mediate colonization, survival and spread in the host [33]. Our results suggest that the selected AI-2 inhibitors, with the exception of C4, might interfere with synthesis, secretion, and/ or transport of AI-2 via their effect on the *luxS*; affecting QS and cognate pathogenicity of APEC [34]. However, C4 also inhibited AI-2 production, this suggests that C4 might intervene at various points in the AI-2 production cycle without having a direct effect on *luxS* and/ or *luxS* dependent mechanisms.

In this study, we found that C2- C9 reduced biofilm formation of APEC O78 (Figure 2A). This result was supported by the inhibitory effect of these compounds on biofilm formation- associated genes (Figure 6A) such as 1) *hha*, which also influences persister cell formation and bacterial resistance to antibiotics [35]; 2) *rcsB, ompG* and *wzb* which are involved in colanic acid capsule biosynthesis that protects the cells against contact-dependent growth inhibition and cell division [36,37]; 3) *bolA* and *murD* which are involved in the production of fimbria-like adhesins, curli and colanic acid and regulate cell morphology, cell wall formation, permeability, cell growth and division, motility and peptidoglycan biosynthesis [23,38]; and 4) *csrA* which regulates central carbohydrate metabolism, glycogen synthesis, gluconeogenesis, cell size and surface properties, motility and flagellum biosynthesis [39]. These findings suggest that the identified AI-2 inhibitors possess anti-biofilm effect against APEC O78 likely through the down-regulation of genes associated with adhesion, motility, and capsule synthesis among others [40]. Our results corroborate previous findings that show other QSIs such as furanone C-30 which inhibited biofilm formation in *Streptococcus mutans* through the inhibition of QS-regulated genes [41]. Additionally, bacterial motility plays an important role in virulence, biofilm formation and pathogenicity of bacteria [21]. Therefore, motility is considered as an attractive target for AI-2 inhibitors for preventing and/or blocking the infection process. In our study, C1- C8 inhibited APEC O78 motility (Figure 2B). This result was supported by the inhibitory effect of these compounds on motility-associated genes (Figure 6A) such as *flgN*, *motB* and *fliP*. Previously, motility inhibitors have been used to control *V. cholerae* and *S. typhimurium* which acted through the down-regulation of flagellar synthesis genes [42,43].

It is known that APEC pathogenesis is controlled by a number of virulence factors and inhibition of these virulence factors by AI-2 inhibitors can render APEC non-virulent and attenuate its pathogenicity. Previously, QSIs such as: furanone C-30 has been reported to regulate virulence of *P. aeruginosa* through the reduction of QS-regulated virulence factors such as protease, pyoverdin and chitinase and their associated proteins [44; 44]. Hamamelitannin, QSI acts through the inhibition of RNAIII, part of the *agr* QS system in *S. aureus* [45], while virstatin was reported to inhibit *V. cholerae* virulence factors such as cholera toxin and toxin coregulated pilus [46]. Interestingly, AI-2 inhibitors in our study also down-regulated a group of the virulence associated genes (Figure 6B) such as 1) *fimC*, which plays an important role in APEC adherence to the cells through the regulation of adhesion of type 1 fimbriae [47], 2) iron uptake receptors (*fyuA* and *lucD*) which are involved in biofilm formation [48], 3) *iss* which plays a role in resistance against serum complements [49], and 4) *vat* which regulates the motility, agglutination, and biofilm formation of APEC [50]. This indicated that the AI-2 inhibitors might interfere with bacterial iron acquisition, metabolism, adhesion and invasion, serum survival; thus, modulate APEC infection of host cells. Notably, all AI-2 inhibitors, except C4, reduced intracellular APEC O78 in chicken and/or human macrophages (Figure 4 A,B), and enhanced *G. mellonella* survival and reduced APEC loads in surviving larvae (Figure 5(b)).

**Figure 7.**
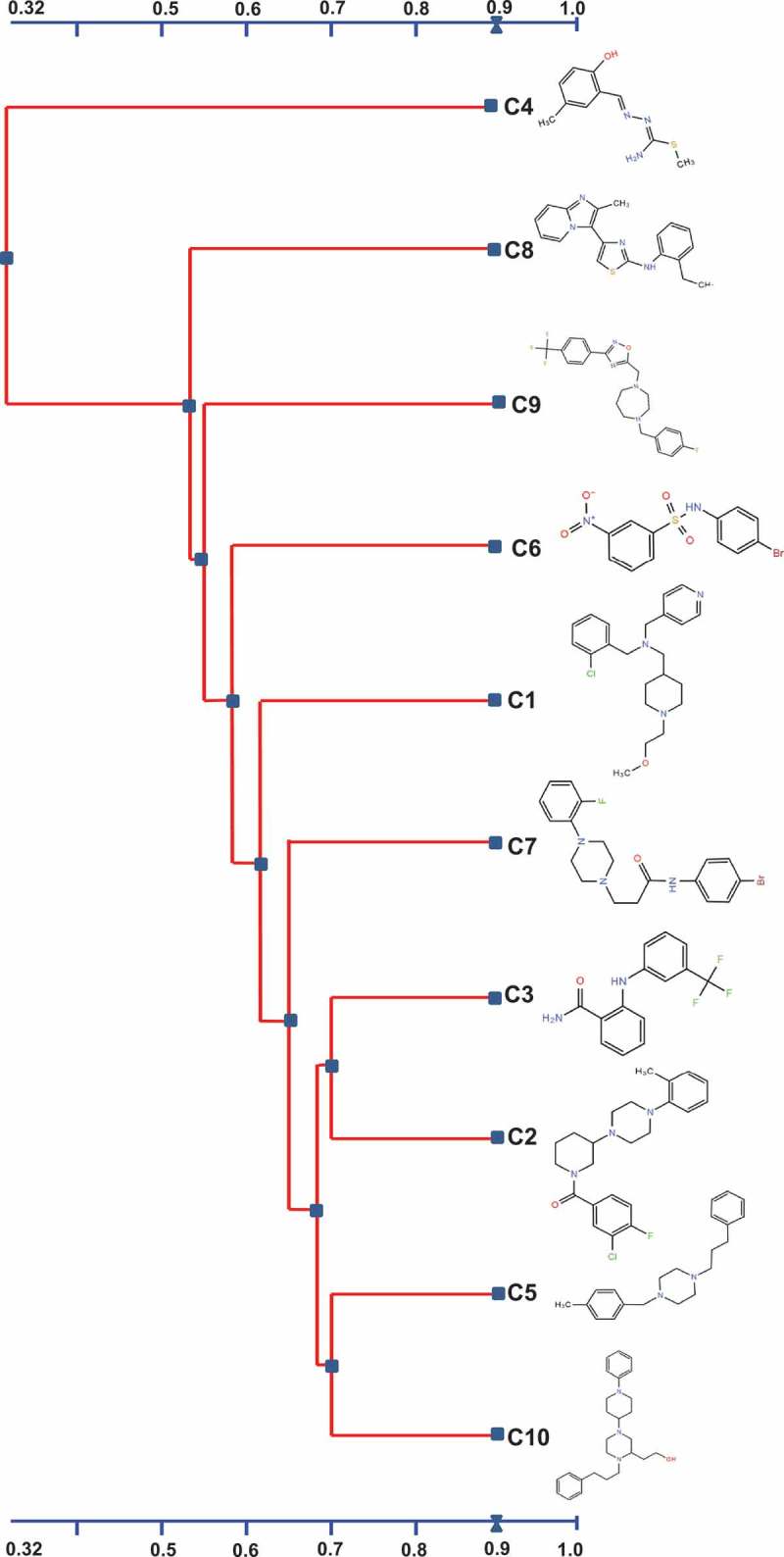
Alignment and the chemical structures of the top 10 AI-2 inhibitors using 2D Tanimoto similarity scoring method. A chemical structure similarity score of 0.68 is statistically significant at the 95% confidence interval.

The two-dimensional structure of the 10 selected AI-2 inhibitors was analyzed using a 2D Tanimoto scoring method [51]. The 10 AI-2 inhibitors were divided into two clusters; the large cluster composed of nine compounds (C1- C3 and C5- C10), while the small cluster was formed only by C4 (Figure 7). All nine compounds within in the larger cluster inhibited the *luxS* expression, while C4 did not, suggesting a potential association between the chemical structure and the mode of action of the compounds in APEC. Four compounds (C2, C3, C5, C10) possess high structural similarities between each other (chemical structure similarity score = 0.71). C5 and C10 are both composed of a phenylpropyl piperazinyl phenyl groups and showed similarity in their effect on expression profile (r > 0.4; P < 0.05, Figure 6A, B) of QS and virulence associated genes. Similarly, both C2 and C10 are composed of a sequence of piperidinyl phenyl piperazinyl groups; however, C2 displayed better *in vitro* effect compared to C10, while C10 displayed better *in vivo* efficacy in wax moths compared to C2 (Figure 5(a)). These findings suggest that the sequence of phenyl piperazinyl functional groups might be key contributors to the AI-2 inhibitory activity of C2, C5 and C10. However, this conclusion requires further experimental analysis.

Although, both C5 and C7 contain piperazinyl functional group (score = 0.58), C6 contains nitrobenzene sulfonamide functional group. However, C6 and C7 have some structural similarity (contain 4- bromophenyl group; score = 0.59), which might explain their similar effect both *in vitro* and *in vivo*. C1, C2 and C10 contain piperidine functional group (score = 0.61) but C1 displayed no effect on biofilm formation (Figure 2A) and lower efficacy *in vivo* compared to C2 and C10 (Figure 5A). This might be due to the presence of piperazine functional group in addition to piperidine functional group in both C2 and C10 suggesting that presence of piperazine functional group might be the key groups contributing to the activity of these compounds (Figure 7). Previously, piperazine based drivatives have been reported to inhibit *V. cholerae* and *P. aeruginosa* through down-regulation of RND efflux virulence and AHL- LasR QS-dependent factors, respectively [52,53].

## Conclusions

The potential AI-2 inhibitors identified in this study differentially affected the pathobiology of APEC and the expression of QS regulated virulence, biofilm, and motility genes. C5, C6, C7 displayed highest efficacy both *in vitro* and *in vivo* and the inhibitory activity seems to be related to piperazine based functional group. Discovery of novel antimicrobials that do not affect bacterial growth is less likely to impose a selective pressure for resistance development by the bacteria and represents an ideal antibiotic independent approach. Further *in vivo* studies in chickens and specific target identification of these potential leads will facilitate development of these AI-2 inhibitors as novel therapeutics to augment APEC control in poultry and prevent its potential zoonotic transmission. Because of their small size and convenient synthesis scheme, we expect the cost of large scale synthesis of these inhibitors to be equivalent or less than the cost associated with synthesis of current antibiotics used in the industry to treat APEC infections. In addition, since human pathogenic ExPECs genetically resemble APEC, our future studies will also test the effect of these inhibitors against human pathogenic ExPECs particularly those that are multidrug resistant.

## Material and methods

### Small molecules library

A library of 4,182 compounds (yactives) were selected via pre-screening of 81,320 compounds and were obtained from Chembridge, Inc. (San Diego, CA, USA) [54]. These compounds were dissolved in 100% dimethyl sulfoxide (DMSO) to a concentration of 10 mM in a 96-well plate and stored at −80 °C for further use.

### Bacterial strains and culture conditions

APEC serotypes O1 (GenBank accession no. NC_008563), O2 (GenBank accession no. NZ_CP006834), and O78 (GenBank accession no. CP004009) were kindly provided by Dr. Tim Johnson (University of Minnesota, Saint Paul, MN). Other APEC serotypes such as O8, O15, O18, O35, O109, and O115 were kindly provided by Drs. Lisa K. Nolan and Catherine M. Logue (University of Georgia, Athens, GA). Luria-Bertani broth (LB; BD Difco) was used for routine propagation of APEC serotypes. APEC serotypes were routinely grown in LB broth overnight at 37 °C with shaking at 200 rpm. Rifampicin resistant APEC O78 (Rif^R^) was isolated by plating the APEC O78 on LB agar containing 50 µg/mL of rifampicin [55], and one spontaneous resistant mutant was used for the wax moth studies. *E. coli* DH5α (Invitrogen) was grown overnight in LB broth at 37 °C with shaking at 200 rpm. *V. harveyi* BB170 (AI-1^+^; AI-2^-^) and *V. harveyi* BB120 (AI-1^+^; AI-2^-^) were kindly provided by Dr. Bonnie L. Bassler (Princeton University) and were grown in AB medium at 30 °C aerobically with shaking as described previously [19].

### Primary screening for non-growth inhibitors

Overnight culture of APEC O78 prepared in LB broth was adjusted to an optical density (OD_600_) of 0.05 (7 × 10^7^ CFU/mL) in fresh LB broth. One hundred micro-liters of the culture was transferred to a 96-well plate and 1µL (100 μM final concentration) of the compound was added to each well using a pin tool [54]. Chloramphenicol (40 μg/mL) or kanamycin (30 μg/mL) and 1μL of 100% DMSO were included as controls in each plate. Plates were incubated at 37 °C for 10 h with shaking in a Sunrise^TM^ Tecan plate reader (Tecan Group Ltd. San Jose, CA, USA) and the growth was kinetically monitored every 30 min by measuring the OD at 600 nm. Compounds that resulted in no growth inhibition (no elevated OD) were chosen for screening in AI-2 bioluminescence inhibition assay.

### AI-2 bioluminescence indicator assay

The AI-2 bioluminescence assay was performed as described previously [2,18,19]. Briefly, APEC O78 cultures grown in the presence of SMs (see above) were centrifuged at 5000 × g for 10 min, and cell-free culture supernatants were prepared by using a 0.22 µm filter 96 well plate (Millipore). The bioluminescence reporter *V. harveyi* BB170 (AI-1^+^ and AI-2^-^) was grown overnight in AB medium at 30 °C aerobically with shaking [19]. The overnight culture was diluted to 1:5000 in fresh AB medium and incubated at 30 °C for 3 h. Following incubation, 180 μL of the *V. harveyi* BB170 culture was distributed into each well of a 96-well plate and mixed with 20 µL of the cell free culture supernatant, the plate was then incubated at 30 °C for 2.5 h in the dark and the bioluminescence was measured using in vivo imaging system (IVIS Lumina Series III, PerkinElmer, USA). The incubation time was determined based on the preliminary studies which showed optimal induction of bioluminescence at 2.5 h (data not shown). Cell-free culture supernatants collected from overnight cultures of *V. harveyi* BB120 (AI-1^+^andAI-2^+^) and *E. coli* DH5α (a strain lacks AI-2) were used as controls [2,19]. Bioluminescence of SMs treated culture supernatants were compared to DMSO treated control. The Z-score was calculated to evaluate the quality of the bioluminescence screening [56]. Four independent experiments were conducted for the 69 compounds that inhibited ≥ 75% of the AI-2 mediated bioluminescence and the average inhibition percentage was calculated. Ten compounds that showed highest AI-2 inhibition were selected for further studies. The details of the selected SMs are listed in Table S1.

The selected compounds were also tested for inhibition of AI-2 production in other APEC serotypes such as O1, O2, O8, O15, O18, O35, O109, and O115 that are commonly implicated in colibacillosis using the same procedure described above.

### Biofilm assay

Effect of the selected AI-2 inhibitors on biofilm formation was assessed using the crystal violet (CV) assay [57]. Briefly, APEC O78 was grown in LB broth in the presence of 100 µM of each compound in a 96-well plate at 37 °C for 10 h. The culture was then diluted 1:100 in fresh LB broth. The biofilm assay was performed in a 96-well plate containing 150 µL of the diluted culture and 100 µM of each compound. The plate was then incubated aerobically without shaking at 37 °C for 48 h, washed twice with PBS to remove the non-adherent cells and stained with 200 µL of 0.1% CV in water at room temperature for 10 min. The plate was washed with PBS and biofilm was quantified by measuring the absorbance at 550 nm after solubilizing the CV in 200 μL of 30% acetic acid in water for 15 min. Two independent experiments were conducted in triplicate wells in each experiment.

### Motility assay

The effect of selected AI-2 inhibitors on APEC motility was performed as described previously [2]. Briefly, overnight culture of APEC O78 was adjusted to an OD_600_ of 0.05 and 100 µL was transferred to each well of a 96 well plate and grown in the presence of 100 µM of each compound at 37 °C for 10 h. The culture was then adjusted to an OD_600_ of 0.05 and used for the motility assay. The motility assay was performed in a 48-well plate using semisolid agar media (0.4% LB agar) containing 0.01% tetrazolium chloride and 100 µM of each compound. One microliter of the OD adjusted culture was stabbed onto the middle of the agar and the plate was incubated at 37 °C for 6 h. The motility was assessed by measuring the diameter of the halo zone in comparison to DMSO treated control. Two independent experiments were conducted with duplicate wells in each experiment.

### Lactate dehydrogenase assay

The selected 10 AI-2 inhibitors were evaluated for their toxicity to Caco-2 (ATCC® HTB-37™) using LDH assay [56]. Briefly, Caco-2 cells (1.4 × 10^5^ cells/well) were grown in a 96-well plate in minimal essential medium (MEM) supplemented with 20% fetal bovine serum (FBS; Gibco), 1% non-essential amino acid (NEAA, Invitrogen Life Technologies) and 1mM sodium pyruvate, at 37 °C in a humidified 5% CO_2_ incubator for 48 h until a complete monolayer was formed. For LDH assay, cells were washed twice with media containing no FBS/no antibiotics and incubated with 150 µL of fresh media containing 100 μM of each compound at 37 °C for 24 h in a humidified 5% CO_2_ incubator. Fifty microliters of supernatant was analyzed using LDH Cytotoxicity Assay Kit (Thermo Scientific). Two independent experiments were conducted in triplicate wells in each experiment.

Toxicity was also determined on HD-11 cells (CVCL-4685). The HD-11 cells (1.4 × 10^5^ cells/well) were grown in Iscove’s modified Dulbecco’s medium (IMEM; Gibco) supplemented with 2 mM glutamine and 10% FBS at 37 °C in a humidified 5% CO_2_ for 48 h until a monolayer was completely formed. LDH assay was conducted as described above. Two independent experiments were conducted in triplicate wells in each experiment.

### Hemolysis assay

The hemolytic activity of the selected AI-2 inhibitors was determined as described before [56]. Briefly, 200 µL of the 10% RBCs (LAMPIRE Biological Laboratories) suspension in PBS was incubated with 100 μM of each compound for 1 h in a 96- well plate. The plate was then centrifuged at 5000 × g for 5 min, placed on ice for 5 min and the absorbance of the supernatant was measured at 540 nm. PBS, 1% DMSO, and 0.1% Triton X-100 were used as controls. Two independent experiments were conducted in triplicate wells in each experiment.

### Intracellular survival assay

The effect of the selected AI-2 inhibitors on the survival of APEC in macrophage cells was tested as described before [58]. Briefly, HD-11 cells was grown as described above and infected with 1 × 10^7^ CFU (MOI = 100) of mid-log phase APEC strains (O78, O1 and O2) at 37 °C for 1 h. Cells were washed with PBS and treated with gentamicin (150 μg/mL) for 1 h to kill the extracellular bacteria [59]. The cells were then washed and incubated with 100 µM of each compound in media containing 10 μg/mL of gentamicin at 37 °C for 6 h. Following treatment, cells were washed, lysed with 0.1% Triton X-100 for 5 min, ten-fold serially diluted in PBS and plated on LB agar plates to determine CFUs. Cells treated with chloramphenicol (40 μg/mL) and 1 μL of 100% DMSO were used as controls. The experiment was conducted two times with four replicates in each experiment. Intracellular survival was expressed as the log change of APEC CFUs in the AI-2 inhibitors treated cells.

Similarly, the intracellular survival assay was also performed in THP-1 cells (ATCC® TIB-202™) as described above. The THP-1 cells were grown in RPMI 1640 medium (Gibco) supplemented with 10% FBS and 2 mM glutamine. In order to differentiate the THP-1 monocyte to macrophage, 100 nM phorbol myristate acetate (PMA; Sigma-Aldrich) was added to the media. The cells (1.4 × 10^5^ cells/well) were grown in a 96-well plate in a 5% CO_2_ incubator at 37 °C for 48 h, infected with APEC (MOI = 100), treated with each compound and the intracellular bacteria was determined as above.

### Wax moth (G. mellonella) larva infection model

*G. mellonella* caterpillars (larvae) in the final instar stage (fifth instar) were obtained from Vanderhorst, Inc. (St. Mary’s, Ohio, USA), stored in wood shavings in a petri dish in the dark and used within 7 days of receiving. Larvae with 15–25 mm length, 250–350 mg weight, having a creamy color with minimal speckling and no grey markings were used in this study. The infection was performed as described previously [28,60]. For the inoculation, AI-2 inhibitors were diluted in a buffer mix (30% DMSO plus 10 mM MgSO_4_) as described previously and each larva (n = 10) was inoculated with 8.5 µL (50 mg/kg; 12.5 µg/larva) of the AI-2 inhibitors into the hemocoel via the last left proleg using PB600-1 repeating dispenser (Hamilton) attached to insulin syringe (31 gauge, 8 mm needle length; ReliOn). Larvae were placed inside sterile petri dishes and incubated for 2 h in the dark at 37 °C. Then, larvae were infected with 8.5 µL of (4.25 × 10^4^ CFU) of Rif^R^ APEC O78 in 10 mM MgSO_4_ on the right hind proleg. Rif^R^ APEC O78 was generated as described above by plating APEC on LB agar plate containing 50 µg/ mL rifampicin for specific monitoring of APEC population inside the larvae. The AI-2 inhibitors exhibited similar AI-2 inhibition against Rif^R^ APEC O78 as that of parent wild-type APEC O78 (data not shown). Infection dose of Rif^R^ APEC O78 to larvae was identified based on preliminary study (Figure S1). Larvae were incubated at 37°C in the dark and the survival was monitored daily for 72 h. Infected larvae treated with buffer mix and 75 mg/kg of chloramphenicol were used as controls.

For the quantification of APEC inside the larvae, dead and live larvae were surface sterilized with 70% ethanol, homogenized in PBS. The suspension was tenfold serially diluted and plated on MacConkey agar plates supplemented with 50 µg/mL of rifampicin. The plates were then incubated overnight at 37 °C and APEC load was enumerated. Two independent experiments were conducted using larvae (n = 10) obtained in different batches.

### Toxicity of SMs in wax moth larvae

To confirm that the death of the larvae was not due to the compounds, intrinsic toxicity the AI-2 inhibitors were assessed in a separate experiment. *G. mellonella* larvae (fifth instar; n = 10) were inoculated with 8.5 µL of SMs (50 mg/kg body weight; 12.5 µg/ larva) into the hemocoel via the last left proleg. Post inoculation, larvae were placed inside sterile petri dishes and incubated in the dark at 37 °C for 72 h and larval survival was monitored every 24 h. Two independent experiments were conducted

### Quantitative real-time reverse transcription PCR (qRT-PCR)

The effect of the selected AI-2 inhibitors on the expression of QS-regulated and virulence factors genes of APEC was determined using qRT-PCR. APEC O78 was grown in the presence of 100 µM of each compound in a 96-well plate at 37 °C for 10 h. APEC O78 culture treated with 1µL of 100% DMSO was used as control. Total RNA was extracted from duplicate wells for each compound (200 µL) using a miRNeasy Mini Kit (Qiagen). RNA quality and quantity was determined by nanodrop 2000 C spectrophotometer (Thermo scientific). Traces of DNA were removed using Genomic DNA removal mix (Qiagen). Approximately, 5 μg of purified RNA was used to synthesize cDNA using the Qiagen RT^2^ First Strand Kit (Qiagen). The qRT-PCR was performed using SensiMix^TM^ SYBR® Hi-ROX qPCR Master Mix (Bioline) according to manufacturer instructions in a realplex^2^ mastercycler (Eppendorf) with 55 ^o^C annealing temperature. Gene-specific primers were designed using PrimerQuest Tool and obtained from integrated DNA technologies (IDT). The primers used with target genes’ description are listed in Table 2. The data were normalized to the house-keeping gene, glyceraldehyde-3-phosphate dehydrogenase (GAPDH) [2]. The relative fold change was calculated using the ∆∆Ct method [61]. Three independent experiments were conducted.

### Statistical analysis

Data from biofilm formation, motility, qRT-PCR, toxicity, hemolytic activity, intracellular survival, and wax moth infection assays were expressed as the mean ± standard deviation. ANOVA followed by Tukey test was used to analyze these data and a P-value < 0.05 was used to determine statistically significant differences between means. A fold change of ± 1.5 ≥ or ≤ 1.5 and a P-value ≤ 0.05 were used to determine statistically significant differences in gene expression. The differences between the gene expression profiles were analyzed using a principal component analysis (PCA) on JMP PRO 13 software (SAS Institute). Statistical analyses of the wax moth infection studies were performed using GraphPad Prism 5 software (GraphPad, Inc.) and plotted using the Kaplan-Meier graph.
