# Supplementary material for: Novel small molecule modulators of quorum sensing in avian pathogenic Escherichia coli (APEC)
Source: Virulence. 2018 Nov 2;9(1):1640–57. doi: 10.1080/21505594.2018.1528844 (PMC7000209; doi:10.1080/21505594.2018.1528844)
Supplement: Supplemental Material [file kvir-09-01-1528844-g0040.zip › APEC-_Supp_material-5.23.18.pdf]

Table S1: Chemical information about the selected AI-2 inhibitors

SM	Chemical name	Molecular weight
C1	(2-chlorobenzyl){[1-(2-methoxyethyl)-4-piperidinyl]methyl}(4-pyridinylmethyl)amine	387.9
C2	1-[1-(3-chloro-4-fluorobenzoyl)-3-piperidinyl]-4-(2-methylphenyl)piperazine	415.9
C3	2-{[3-(trifluoromethyl)phenyl]amino}benzamide	280.2
C4	methyl N'-(2-hydroxy-5-methylbenzylidene)hydrazonothiocarbamate	223.3
C5	1-(4-methylbenzyl)-4-(3-phenylpropyl)piperazine	308.5
C6	N-(4-bromophenyl)-3-nitrobenzenesulfonamide	357.2
C7	N-(4-bromophenyl)-3-[4-(2-fluorophenyl)-1-piperazinyl]propanamide	406.3
C8	N-(2-ethylphenyl)-4-(2-methylimidazo[1,2-a]pyridin-3-yl)-1,3-thiazol-2-amine	334.4
C9	1-(4-fluorobenzyl)-4-({3-[4-(trifluoromethyl)phenyl]-1,2,4-oxadiazol-5-yl}methyl)-1,4-diazepane	434.4
C10	2-[4-(1-phenyl-4-piperidinyl)-1-(3-phenylpropyl)-2-piperazinyl]ethanol	407.6

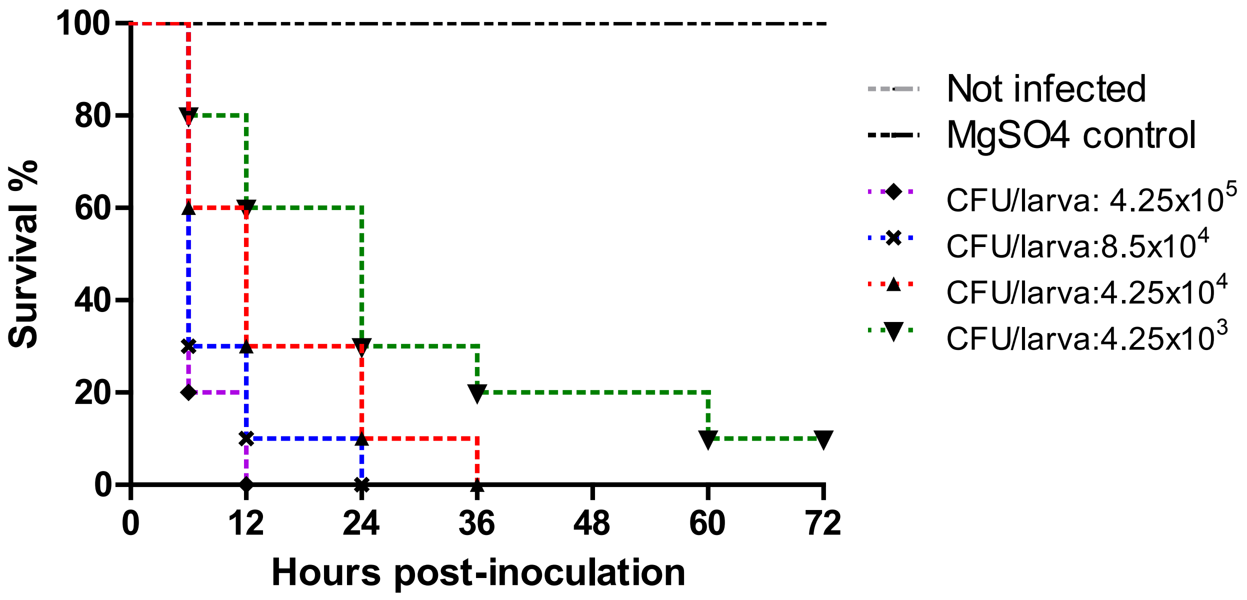

Figure S1: Determination of the Rif^r APEC O78 infection dose to the wax moth larvae. Each larva (n= 10) was inoculated with 8.5 µL of different concentration of Rif^r APEC O78 grown to the mid exponential phase via the last right proleg. Larvae were incubated at 37 ^{0}C in the dark and the survival was monitored for 72 h. For the quantification of APEC inside the larvae, dead and live larvae were surface sterilized with 70% ethanol, homogenized in PBS and tenfold serial dilutions of the suspension was plated on MacConkey agar plates supplemented with 50 µg/mL of rifampicin. The plates were then incubated overnight at 37 ^{0}C and CFUs determined.
